# Caring for the caregivers: a scoping review of spiritual and religious interventions for family caregivers in home care

**DOI:** 10.1186/s12912-026-05173-y

**Published:** 2026-08-03

**Authors:** Jenny Kubitza, Benedikt Kaiser, Ruth Mächler, Dagmar Teutsch, Juliane Stingl, Annette Haußmann, Eckhard Frick

**Affiliations:** 1 TUM School of Medicine and Health, Department of Psychosomatic Medicine and Psychotherapy, Spiritual Care and Psychosomatic Health, Technical University of Munich, TUM University Hospital, Munich, Germany; 2 Faculty of Social Work, Baden-Wuerttemberg Cooperative State University (DHBW), Villingen-Schwenningen, Germany; 3 Faculty of Theology, Ruprechts-Karls-University Heidelberg, Heidelberg, Germany

**Keywords:** Caregiving needs, Domestic care, Informal caregiving, Outpatient care, Religion, Spirituality

## Abstract

**Background:**

Family caregivers often experience stress, which negatively affects their well-being, health, and quality of life. This review of current studies on spiritual and religious interventions and their effects will contribute to the development of holistic strategies to support family caregivers.

**Methods:**

This scoping review followed Arksey and O’Malley’s framework and is reported in accordance with the modified Preferred Reporting Items for Systematic Reviews and Meta-Analyses Extension for Scoping Reviews (PRISMA-ScR) guideline. A systematic search of studies and grey literature examining spiritual and/or religious interventions for family caregivers in the home setting was conducted across nine databases and Google Scholar.

**Results:**

The search yielded 608 hits, of which 19 studies met the inclusion criteria; a total of 1,025 family caregivers are involved. Most studies have a quantitative design, examine the feasibility and pilot testing of the interventions, and are of moderate quality. Interventions include spiritual training and therapy, faith community nursing, yoga, meditation, mindfulness programs, and self-expressive therapies. These range from single-session approaches to programs lasting several weeks and are conducted as individual, dyadic, or group sessions, either in-person, by telephone, or via virtual platforms. Most studies report improvements in spiritual well-being, health, and quality of life. Some studies reported deterioration in physical health and increased fatigue following spiritual or religious interventions.

**Conclusion:**

Spiritual or religious interventions can reduce stress among family caregivers and improve their well-being, provided they are tailored to their needs. To maximize their benefit, interventions should be tailored to caregivers’ needs, be flexible in timing and easily accessible, foster meaningful relationships, and be of sufficient duration to enable them to develop practical strategies for daily life.

**Supplementary Information:**

The online version contains supplementary material available at 10.1186/s12912-026-05173-y.

## Background

Family caregivers (FCs) are defined as individuals who provide unpaid care for relatives or friends, assisting with physical, cognitive, or emotional aspects of life on a daily or intermittent basis, often over extended periods, with a high level of responsibility [1]. Around the world, they typically provide most of the day-to-day support for people with chronic or serious illnesses [2–5], including assistance with household tasks, transportation, mobility, self-care, medication management, and healthcare-related activities [5].

FCs are often exposed to various stressors and often neglect their own health [6–8]. For example, in a study of 27,000 FCs, two-thirds report recovering little or not at all from stress, and only one-third regularly pursue their own interests. These FCs describe their situation as a constant „need to function“ [7].

Besides the physical and psychological demands, the spiritual dimensions of caregiving are gaining increasing attention. Spirituality is commonly understood as a dynamic connection encompassing the search for meaning and purpose in life. This connection may involve a higher power (as God), something transcendent, relationships with others, the self, and/or nature [9]. It has been described as a human being’s property that can foster processes of healing and reconciliation and support individuals in coping with life challenges, often enabling the attribution of positive meaning to difficult experiences [10, 11].

Although spirituality is often expressed through religious beliefs and practices, the two concepts are not synonymous. Spirituality generally refers to an individual‘s search for meaning, purpose, and connection, which may occur within or outside a religious framework. In contrast, religion is typically characterized by organized systems of beliefs, values, traditions, and practices shared within a faith community and may include activities such as prayer, worship, rituals, and engagement with sacred texts. Nevertheless, both constructs are closely related and frequently overlap, with religious beliefs and practices serving as important expressions of spirituality [12, 13]. Furthermore, both spirituality and religion are culturally embedded concepts whose meanings may vary across social and cultural contexts. For the purpose of this review, spirituality is understood as an overarching concept encompassing both religious and non-religious forms.

Among FCs, spirituality has been identified as a relevant coping resource associated with better health and quality of life [14]. Research suggests that spirituality supports self-reflection and mindful awareness of personal needs and boundaries. It can also facilitate meaning-making in caregiving, including recognizing potential positive aspects of home care and accepting changed life circumstances [15–18].

At the same time, spiritual coping is not always beneficial. Two qualitative longitudinal studies indicate that FCs can experience spiritual strain when they are unable to find meaning in their situation or when caregiving is interpreted as injustice or punishment [18, 19]. These findings highlight the ambivalent nature of spirituality in caregiving contexts, where it may function as both a resource and a source of stress.

Despite its relevance, spiritual support for FCs remains insufficiently integrated into routine care [18, 19]. A preliminary search of the Cochrane Database of Systematic Reviews did not identify any review on spiritual or religious interventions among FCs.

Given the increasing burden on FCs, there is a need for holistic support strategies that include spiritual resources and coping strategies. To inform future development and practice, an overview of the current evidence on spiritual and religious interventions in FCs is required. Therefore, this study aims to systematically map and synthesize the current evidence on spiritual and religious interventions for FCs.

## Methods

### Study design

This study adopts a scoping review design that involves the mapping of literature and scientific literature without evaluating the evidence. This review was conducted following the methodological framework proposed by Arksey and O’Malley [20]. The review is reported in accordance with the Preferred Reporting Items for Systematic Reviews and Meta-Analysis Extension for Scoping Reviews (PRISMA-ScR) guideline developed by Tricco et al. [21]. Consistent with Arksey and O´Malley´s framework, the review comprises five steps: (1) identifying the research question, (2) identifying relevant studies, (3) study selection, (4) charting the data, and (5) collating, summarizing, and reporting the results. The methodical procedure is described in more detail in the protocol [22].

### Identifying the research question(s)

The main review question is: What evidence exists on spiritual and religious interventions that support FCs in coping with the caregiving situation in a home setting? More specific review questions are as follows:

1. Which FCs in the home setting have been included?
2. How are these interventions structured, and what content is covered?
3. What outcomes have been reported?

### Identifying relevant studies

CINAHL, Cochrane Library, JBI Evidence Synthesis, MEDLINE via PubMed, PSYNDEX, PsycINFO, Scopus, Springer Link, and Web of Science were searched from November 2025 to December 2025. To ensure that all relevant information was included, the grey literature and unpublished studies were searched using Google Scholar, and the included articles were tracked for citations.

Prior to the main search, a pilot search was conducted on MEDLINE to pre-select keywords from the titles and abstracts of papers considered relevant to the subject. The search strategy combined key concepts using the PCC framework (population, context, concept), keywords from relevant articles, and MeSH terms (Table 1). It was discussed with six researchers until consensus was reached (AH, DT, EF, JK, KZ, RM). The full search strategy was used in all databases and adapted where necessary (Supplement file 1). For the grey literature search, Google Scholar was searched using the same search strategy applied in the databases. The first ten pages of results sorted by relevance were screened. The search was limited to articles published between 2015 and 2025 and written in English, French, German, Italian, or Spanish.

**Table 1:** Key concepts, key words, and MeSH terms

PCC	Search terms and synonyms	MeSH terms MEDLINE
Population	(family caregiver* OR informal caregiver*)	Family caregiver, Informal caregiver, Spouse caregiver, Caregivers
Context	(home OR domestic OR outpatient)	Home care
Concept	(spiritual* OR religio* OR hop* OR faith OR meaning OR purpose OR existent* OR transcendence* OR connect* OR aware* OR confidence OR creativ* OR art OR music) AND (intervention* OR cop* OR support OR counsel* OR training* OR education OR instruction*)	Spirituality, Spiritualities, Spiritual therapies, Religion, Faith Healing, Hope, Art, Music, Coping skills, Education

### Study selection

One reviewer (JK) combined and entered all database searches into EndNote (20.6/2023). After removing duplicates, the hits were uploaded to Rayyan, a systematic review management platform. First, two reviewers (BK, JK) independently screened the titles and abstracts against the eligibility criteria, followed by the full texts. Records identified through Google Scholar were screened in the same manner, with the title and abstract screening followed by full-text assessment according to the predefined eligibility criteria. In case of doubt, both reviewers discussed until a mutual decision was reached. The studies were provided to the research team for reading and rating (BK, DT, EF, JK, JS, KZ, RM).

The eligibility criteria were also based on the PCC framework and the study designs (Table 2). Sources were included that examined family or informal caregivers in home care who received spiritual and/or religious interventions. Sources included published research from qualitative, quantitative, and mixed-methods studies. Studies were excluded if they were reviews or meta-analyses, editorials, points of view, letters to the editor, correspondences, or interviews. As the focus was on primary data rather than other researchers’ interpretations, all reviews were excluded from the analysis. Relevant reviews on the topic are therefore only included in the later discussion, not in the results.

**Table 2:** Inclusion and exclusion criteria

PCC	Inclusion criteria	Exclusion criteria
Population and Context	Studies on family caregivers or informal caregivers in home care who require or don´t require professional support	Studies on family caregivers or informal caregivers in inpatient facilities
Concept	Studies evaluating interventions explicitly described as spiritual, religious, or spiritual/religious	Studies evaluating interventions not explicitly described as spiritual, religious, or spiritual/religious
Study design	Qualitative studies	Reviews
	Quantitative studies	Meta-analysis
	Mixed Methods	Editorials
		Points of view
		Letters to editors
		Correspondences
		Interviews

### Charting the data

To ensure accuracy, the studies were analyzed independently and then compared between two researchers (BK, JK) to reach consensus. For the analysis, the authors used a data-extraction sheet to collect data on publication date, country, discipline, research question, study design, measures, sample size, sample characteristics, and design/ content/ effectiveness of the intervention.

The study design was assessed by seven independent researchers (BK, DT, EF, JK, JS, KZ, RM) using the Standard Quality Assessment Criteria [23]. The first or second author (BK or JK) and one other team member read and rated each study. Each study was presented to the full research team, with the assigned rating explained and justified, followed by discussion until consensus was reached. The score sheet for qualitative studies consists of ten items, while the one for quantitative studies consists of 14 items. As each researcher could rate the items with a maximum of two points, the single items score with a maximum of four points, resulting in the highest possible total score of 40 points for qualitative studies (2 reviewers x 10 items x 2 points), and 56 points for quantitative studies (2 reviewers x 14 items x 2 points). Mixed-methods studies were scored in both categories. Next, overall scores were calculated for each study and standardized as a percentage of the maximum possible score [23]. Because scoping reviews are exploratory by nature, no studies were excluded based on quality criteria.

### Collating, summarizing, and reporting the results

The results of the qualitative studies were synthesized using thematic synthesis. To this end, the texts were coded line by line based on the research questions, and common themes were synthesized [24]. The quantitative studies were summarized descriptively and combined with the synthesized qualitative data in accordance with the research questions. The results were presented in narrative or visual formats, regardless of their methodological quality.

## Results

**Fig. 1:**
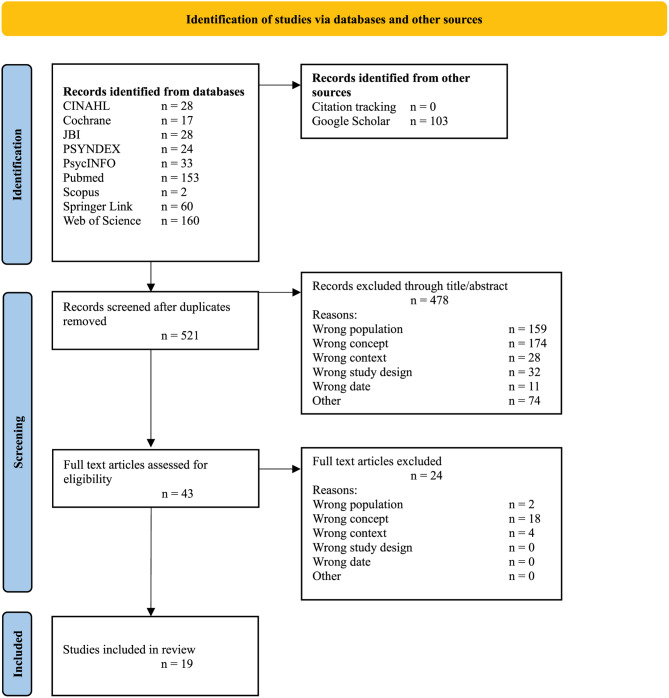
PRISMA flow diagram

The search yielded 608 records. After removing duplicates and reviewing the results against the established criteria, 19 studies are included in the review. The criteria for study exclusion are explained in the PRISMA flow diagram (Fig. 1).

### Characteristics of the selected studies

The 19 studies are published between 2015 [25] and 2025 [26, 27]. Among the 19 studies, most use quantitative research methods (*n =* 16), whereas two are mixed-methods [28, 29], and one is qualitative [30]. Two studies describe their design as quantitative but supplement their data collection with post-intervention interviews [31, 32]. Most of the studies are conducted in the U.S. (*n =* 12), the remaining studies are conducted in Iran (*n =* 4) [26, 33–35], China (*n =* 1) [26], Portugal (*n =* 1) [29], and Spain (*n =* 1) [36]. Research on this topic is being conducted in the fields of nursing (*n =* 10), palliative care and oncology (*n =* 3), psychology (*n =* 2), medicine (*n =* 1), public health (*n =* 1), and narrative self-expression (*n =* 1). Table 3 shows an overview of the studies’ characteristics.

**Table 3:** Summary of relevant studies (*n =* 19)

Authors (Year)	Country	Discipline	Research question	Study design and Method	Measures	Sample and Sample size	Intervention	Key outcomes/ effectiveness
Bagheri et al. [26] (2025)	IRN	Nursing	How does a 4-week spiritual training affect self-efficacy and stress levels of FCs?	2-arm randomized clinical trial with a pre- and follow up-survey	Caregiver Self Efficacy Questionnaire	Mothers of children with leukemia	Spiritual Care guidelines of the Sound Heart Model	Significant increase in self-efficacy;
					Caregiver Burden Inventory	*n* = 64	5 individual sessions (45 min each) with guided interviews and educational sessions	Significant reduction in caregiver burden
							Arm 1: intervention	
							Arm 2: no intervention	
Beauchamp et al. [37] (2023)	USA	Nursing	How feasible is a 4-week meditation intervention and what are the effects on the symptoms of depression, anxiety, and pain of FCs?	2-arm randomized clinical trial with a pre-, post-, and follow up-survey	Feasibility Data	Adult FCs of stroke survivors	Tibetan Three Doors tradition	Significant reduction in anxiety within-group;
					Center for Epidemiologic Studies Depression Scale	*n* = 23	4 group sessions (60 min each) with instructions and exercises	No effect on depression within-group;
					State-Trait Anxiety Inventory for Adults		Arm 1: intervention	No between-group effects;
							Arm 2: no intervention	
								Low adherence
Casaleiro et al. [29] (2024)	PRT	Nursing	How does a 6-week spiritual intervention affect spiritual coping, quality of life, and the burden of FCs?	Mixed-methods 1-arm pilot trial with a pre- and post-survey	Brief Religious Coping Scale	Adult FCs of relatives with severe mental illness	Modified intervention from Oncologist Assisted Spiritual Intervention Study	Improved spiritual coping, mental health, and quality of life;
				Content analysis from the open-ended question	Short Form Health Survey SF-12v2	*n* = 10	3 individual sessions (60 min each) with guided interviews	Deteriorations in physical health;
					Informal Caregiver Burden Assessment Questionnaire			High perceived relevance and acceptability
					Open-ended question about experiences during the intervention			
Diaz-Rodriguez et al. [36] (2021)	ESP	Nursing	How does a 4-week meditation program affect psychological outcomes and cardiovascular/ autonomic nervous system balance of FCs?	1-arm clinical trial with a pre- and post-survey	Lima Happiness Questionnaire	High-burden FCs	Focused Attention Meditation program	Significant increase in positive sense of life, satisfaction in life, personal realization, happiness of living, and some HRV parameters;
					Hospital Anxiety and Depression Scale	*n* = 37	2 group sessions (120 min each) with educational sessions and exercises	Significant reduction in anxiety, blood pressure, and resting heart rate;
					Heart Rate Variability			No effect on depression
					Blood pressure			
					Resting heart rate			
Kaimal et al. [28] (2020)	USA	Art therapy	How does collage art-based affect stress, spiritual wellbeing, self-efficacy, cortisol, and oxytocin of FCs compared to narrative self-expression?	Mixed-methods 2-arm quasi-experimental trial with a pre- and post-survey	Positive and Negative Affect Schedule	Adult FCs of patients at the end of life	Narrative self-expression	Improved positive emotions;
				Thematic analysis from the collages and interview notes	General Self Efficacy Scale	*n* = 14	1 individual 60-min session with a collage and guided interview	Perceived improvements in
					Spiritual Well-being Scale			recognizing burdens and reflections on purpose/values;
							Arm 1: collage art therapy	Reduced spiritual well-being;
					Perceived Stress Scale		Arm 2: narrative self-expression	
					Markers of stress (cortisol, oxytocin)			No effect on reducing negative emotions, cortisol, and oxytocin
					Collages and interview notes			
Kazmer et al. [30] (2018)	USA	Nursing	What are the experiences of FCs and faith community nurses participating in a 6-months faith community nurse–led cognitive-behavioral and spiritual counseling intervention?	Qualitative longitudinal study (4 surveys)	Semi-structured interviews	Adult FCs of relatives with dementia	Faith Community Nurse-led Cognitive Behavioral and Spiritual Counseling	Perceived improvements in coping and self-care awareness;
				Content Analysis of the interviews		*n* = 7	12 individual sessions (60 min each) with guided interviews and educational sessions	Initial skepticism, but high acceptability after trust development
Kubo et al. [31] (2019)	USA	Public Health	How feasible is an 8-week mHealth mindfulness intervention and what are the effects on distress and quality of life of FCs?	2-arm randomized pilot trial with a pre- and post-survey	Feasibility Data	Adult FCs of patients with cancer	Mindfulness app ‘Headspace’	Significant increase in mindfulness;
				Thematic Analysis of the interviews	NCCN Distress Thermometer	*n* = 31	56 individual sessions (20 min each) with guided exercises and educational sessions	Improved posttraumatic growth and personal strength;
					Hospital Anxiety and Depression Scale		Arm 1: intervention	No effects on distress, anxiety, depression, sleep, pain, fatigue, and quality of life;
					PROMIS Pain Intensity & Pain Interference		Arm 2: no intervention	High acceptability, but adherence challenges
					PROMIS Sleep Disturbance			
					Caregiver Quality of Life Index-Cancer			
					Brief Fatigue Inventory			
					Five Facet			
					Posttraumatic Growth Inventory			
					Mindfulness Questionnaire Short Form			
					Post-Intervention Interview			
Leng et al. [38] (2024)	USA	Medicine	How does a 12-week yoga intervention affect symptoms of depression and quality of life?	1-arm pilot trial with a pre- and post-survey	Patient Health Questionnaire	Adult FCs of patients with lung cancer	Vinyasa Style Yoga program	Significant increase in quality of life;
					Caregiver Quality of Life Index-Cancer	*n* = 23	36 couple-based sessions (60 min each) with (pre-recorded) instructions and exercises	Improved physical activity;
					The Godin Leisure Time Exercise Questionnaire			Significant reduction in depression;
					Dyadic Adjustment Scale			Reduced fatigue;
					Multidimensional Caregiver Strain Index			No effects on caregiver burden
					Multidimensional Scale of Perceived Social Support			
Mahdavi et al. [34] (2017)	IRN	Nursing	How does a 5-week spiritual group therapy affect strain of FCs?	3-arm experimental trial with a pre- and post-survey	Robinson’s Caregiver Strain Index	Adult FCs of elderly patients with Alzheimer	Richards and Bergin’s spiritual model	Significant reduction in caregiver burden
						*n* = 89	5 group sessions (60 min each) with exercises, discussions, and reflections	
							Arm 1: intervention	
							Arm 2: group sessions without interventions	
							Arm 3: no intervention	
Milbury et al. [25] (2015)	USA	Oncology Palliative	How does a 6-week couple-based yoga intervention affect fatigue, sleep disturbances, distress, quality of life, spirituality, and relational closeness of patients and their FCs?	1-arm pilot trial with a pre- and post-survey	Feasibility Data	Adult FCs of patients with non-small cell lung cancer	Vivekananda Yoga and Tibetan Yoga program	Improved spiritual well-being and physical aspects of quality of life;
					Brief Symptom Inventory-18	*n* = 9	15 group sessions (60 min each) with instructions, exercises, and printed material	Significant reduction in sleep disturbances;
					Pittsburgh Sleep Quality Index			Reduced depression;
					Brief Fatigue Inventory			Moderate adherence, but high satisfaction and frequent home practice
					Medical Outcomes Study 36-item short-form survey			
					Spiritual Well-Being Scale			
					Benefit Finding in Cancer Scale			
					Laurenceau’s perceived Closeness and Responsiveness Measure			
Milbury et al. [39] (2018)	USA	Oncology Palliative	How feasible is a 6-week couple-based yoga intervention for FCs?	1-arm pilot trial with a pre- and post-survey	Feasibility Data	Adult FCs of patients with high-grade glioma	Vivekananda Yoga program	Improved mental health and depression;
					Brief Fatigue Inventory	*n* = 5	15 group sessions (60 min each) with instructions, exercises, printed material, and a DVD with the entire program	Deteriorations in physical health, sleep quality, and fatigue;
					Centers of Epidemiological Studies Depression Scale			
					MDASI Health Component Summary			High adherence and full completion, high acceptability and regular independent practice
					Pittsburgh Sleep Quality Index			
Milbury et al. [32] (2022)	USA	Oncology Palliative	How feasible is a 6-week couple-based yoga intervention and a single-based yoga intervention compared to usual care for FCs?	3-arm randomized controlled trial with a pre-, post-, and follow up-survey	Centers of Epidemiological Studies Depression Scale	Adult FCs of patients with glioma	Hatha Yoga program	Improved mental quality of life;
					Medical Outcomes Study	*n* = 67	15 group sessions (45 min each) with instructions and exercises	Reduced caregiver burden;
					Caregiver Reaction Assessment		Arm 1: couple-based	No effects on depression and physical quality of life;
					Interview to Feasibility and Acceptability		Arm 2: single-based	Moderate adherence, better engagement in individual format
							Arm 3: no intervention	
Perez et al. [40] (2022)	USA	Public Health	How feasible is a chaplain-delivered spiritual care assessment and intervention (SCAI) for patients and their FCs?	1-arm pilot trial with a pre-, post-, and follow-up survey	Functional Assessment of Chronic Illness Therapy-Spiritual Well-Being Scale	Adult FCs of patients with advanced lung or gastrointestinal cancer	Spiritual Care Assessment and Intervention Framework	Significant improvements in quality of life at 6 weeks and 12 weeks;
		Theology	How does SCAI affect spiritual well-being, quality of life, depression, anxiety, and religious coping of patients and their FCs?		Caregiver Quality of Life Index-Cancer	*n* = 18	4 individual sessions (30 min each) with guided interviews	Improved spiritual well-being and religious coping;
					Generalized Anxiety Disorder Scale			Reduced anxiety and depression
					Patient Health Questionnaire			
					Religious Coping			
Pomeroy et al. [41] (2023)	USA	Nursing	How does a 12-week Faith Community Nursing intervention affect well-being, disease management, advocacy and self-care skills of FCs?	1-arm quasi-experimental trial with a pre-, peri-, and post-survey	The Preparedness for Caregiving Scale	Adult FCs of older patients	Faith Community Nurse Connection	Significant improvement in spiritual well-being, self-esteem, and disease management;
					Model for Healthy Living	*n* = 16	7 couple-based sessions (60 min each) with guided interviews and educational sessions	No effects on healthy living and depression
					Spirituality as Life Meaning, Purpose and Connection with/without religiosity			
					Family Caregiver survey			
Qorbani et al. [33] (2024)	IRN	Nursing	How does a 2-week religious and spiritual care training affect spiritual health and care burden of FCs during the COVID-19 pandemic?	2-arm randomized pilot trial with a pre- and post-survey	Spiritual Health Questionnaire	Adult FCs of elderly people	Richards and Bergin’s spiritual model	Significant improvement in spiritual health;
					Care Burden Questionnaire	*n* = 80	4 individual virtual offline sessions with exercises and reflections	No effects on religious health and depression
							Arm 1: intervention	
							Arm 2: no intervention	
Salamizadeh et al. [35] (2017)	IRN	Nursing	How does a 5-week spiritual intervention affect self-efficacy of FCs?	2-arm quasi-experimental trial with a pre-, and post-survey	General Self Efficacy Scale	Adult FCs of patients with Alzheimer	Intervention developed by Reyhani et al.	Significant improvement in self-efficacy
						*n* = 54	5 sessions (60 min each) with educational sessions	
							Arm 1: intervention	
							Arm 2: no intervention	
Sun et al. [42] (2016)	USA	Nursing	How does a spiritual intervention affect spiritual well-being of FCs?	2-arm quasi-experimental trial with a pre-, and post-survey	City of Hope FCG QOL Tool-Spiritual Well-Being Subscale	Adult FCs of patients with lung cancer	National Consensus Project’s Clinical Practice Guidelines for Palliative Care	Significant improvement in meaning/peace subscale
						*n* = 354	4 individual sessions (30 min each) with guided interviews and educational sessions	
Waelde et al. [43] (2017)	USA	Psychology	How does a 12-week meditation program affect depression, self-efficacy, and stress of FCs?	2-arm randomized pilot trial with a pre- and post-survey	Center for Epidemiological Studies Depression Scale	Older female FCs of patients with Dementia	Inner Resources Meditation program	Significant improvements in life satisfaction;
					Revised Scale for Caregiving Self Efficacy	*n* = 31	11 group sessions a 90 min with instructions and exercises	Significant reduction in cortisol slope;
					Markers of stress (cortisol)		Arm 1: intervention	No effects on depression and self-efficacy
							Arm 2: Psychoeducation and telephone support	
Zhang et al. [27] (2025)	CN	Psychology	How does a 4-week group mindfulness-based intervention affect mental health outcomes in FCs compared to psychoeducation and usual care?	3-arm randomized controlled trial with a pre-, post-, and follow up-survey	Center for Epidemiological Studies Depression Scale	Adult FCs of frail older adults	Mindfulness-Based Stress Reduction and Mindfulness-Based Cognitive Therapy	Significant improvements in family function, self-efficacy, and problem-solving coping;
					Zarit Burden Interview	*n* = 93	4 group sessions (180 min each) with instructions and exercises	Significant reduction in depression and experiential avoidance
					Hospital Anxiety and Depression Scale		Arm 1: intervention	
					Functional Assessment of Chronic Illness Therapy-Spiritual Well-Being Scale		Arm 2: psychoeducation program	
					Family APGAR Scale		Arm 3: no intervention	
					Modified Conflict Tactics Scale			
					Caregiver Inventory			
					Brief COPE			
					Brief Experiential Avoidance Questionnaire			

### Study quality

Overall, the mean quality score is 64.6%, with higher scores in quantitative (70.9%) than in qualitative studies (58.3%). The mixed-methods study by Kaimal et al. provides the lowest quality score (35%) [28], while the highest quality score is provided by the quantitative 3-arm experimental study by Mahdavi et al. (92%) [34]. The remaining studies provide moderate quality scores (50–90%) (Table 4).

The main limitations identified in the qualitative studies are insufficient description and justification of data collection procedures (mean item score = 1.3) and a limited reflexive account of the study process (mean item score = 0.6). The quantitative studies are primarily unable to achieve an appropriate sample size (mean item score = 1.4) and are rarely checked for confounding (mean item score = 2.0). In randomized studies, the blinding of participants (mean item score = 0.8) and researchers (mean item score = 1.1) is neglected. An overview of all items is available in the Supplement files 2 and 3.

**Table 4:** Assessment of the study quality (*n =* 19)

Qualitative Studies		
Study	Total score / 100%	Limitation
Casaleiro et al. [29] (2024)	60%	Incomplete description of the data collection and analysis
		No reflexivity of the account
Kaimal et al. [28] (2020)	35%	Conducting the intervention, data collection, and analysis by a single researcher (art therapist)
Kazmer et al. [30] (2018)	80%	Incomplete description of data collection
		Incomplete reflexivity of the account

Quantitative Studies		
Study	Total score / 100%	Limitation
Bagheri et al. [26] (2025)	79%	Incomplete description of randomization
		Poor blinding of investigators
		No blinding of subjects
Beauchamp et al. [37] (2023)	61%	High dropout and inappropriate sample size
		Poor control of confounding
Casaleiro et al. [29] (2024)	64%	No description of data analysis
		Inadequate sample size
Diaz-Rodriguez et al. [36] (2021)	73%	No control of confounding
		Inappropriate sample size
Kaimal et al. [28] (2020)	55%	No description of subject characteristics Inadequate sample size
		No control of confounding
Kubo et al. [31] (2019)	88%	No blinding of investigators
		Inappropriate sample size
Leng et al. [38] (2024)	45%	Inadequate sample size
		No control of confounding
		No conclusion supported by the results
Mahdavi et al. [34] (2017)	92%	No blinding of subjects
Milbury et al. [25] (2015)	59%	No description of subject characteristics Inadequate sample size
		Incomplete description of data analysis
Milbury et al. [39] (2018)	73%	No correlation between research aims and outcomes
		Incomplete description of data analysis
Milbury et al. [32] (2022)	81%	No blinding of investigators
		Inadequate sample size
Perez et al. [40] (2022)	68%	Inadequate sample size
		No estimate of variance for the main results
Pomeroy et al. [41] (2023)	64%	Inadequate sample size
		Incomplete reporting of results
		Poor control of confounding
Qorbani et al. [33] (2024)	85%	No blinding of investigators
		Poor control of confounding
Salamizadeh et al. [35] (2016)	77%	Inappropriate sample size
		Incomplete description of data analysis
		Poor control of confounding
Sun et al. [42] (2016)	59%	Inappropriate sample size
		Poor correlation between research aims and outcomes
		No conclusion supported by the results
Waelde et al. [43] (2017)	64%	No description of randomization
		No blinding of subjects
		Inadequate sample size
Zhang et al. [27] (2025)	89%	Inadequate sample size
		Poor blinding of investigators

### Participants of the interventions

In total, the 19 studies examine 1,025 FCs in outpatient settings. The sample sizes range from five [39] to 354 [42]. Most studies focus on FCs of cancer patients (*n* = 8) [25, 26, 31, 32, 38, 39], four studies examine FCs of patients with dementia or Alzheimer’s disease [30, 34, 35, 43], three studies examine FCs of frail older adults [27, 33, 41], and one study each examines FCs of stroke survivors [37], patients with mental illness [29], and palliative care patients [28].

All studies survey only adult FCs; otherwise, most studies do not specify any additional inclusion criteria regarding age, gender, or relationship to the patient. As exceptions, Bagheri et al. study mothers [26], Milbury et al. study only married spouses [39], and Waelde et al. study only female FCs [43]. Diaz et al. [36] and Zhang et al. [27] also include only burdened FCs.

The most frequently surveyed participants are married women who are caring for their spouses, partners, or parents. The average age is 55.6 years. The lowest average age is in the study by Bagheri et al., which exclusively examines mothers of children with leukemia (mean age = 36.56 years) [26]. The highest average age is 69 years, observed among FCs of palliative care patients [28].

Six studies mention the religious and/or spiritual affiliations of the FCs [26, 27, 29, 33, 40, 42]. Except for the study by Zhang et al. [27], most [40, 41] or all [26, 27, 33] of the FCs in the other five studies report being religious and/or spiritual.

In addition to the FCs, eight studies also survey the patients [25, 31, 32, 37–40, 42].

### Content and structure of the interventions

Interventions for FCs include (a) spiritual training and therapy [26, 29, 33–35, 40, 42], (b) faith community nursing [30, 41], (c) yoga [25, 32, 38, 39], (d) meditation [36, 37, 43], (e) mindfulness programs [27, 31], and (f) self-expressive therapies [28].

- Spiritual training and therapy:

Seven studies describe their intervention as spiritual training or therapy. The training programs and therapies are based on models [26, 33, 34], frameworks [40], guidelines [42], or studies [29, 35], while only two of the seven studies refer to the same source (Richards and Bergin’s model) [33, 34]. Richards and Bergin’s model primarily involves religious interventions, such as prayers, conversations about divine issues, consulting with religious leaders, and using religious book therapy [33, 34]. The program from Salamizadeh et al. also primarily addresses religious themes, such as trust in God, seeking help from holy people, generosity, altruism, mantras, and prayer [35]. Salamizadeh et al. and Mahdavi et al. structure their programs into five in-person 60-minute group sessions spread over five weeks [34, 35]; Qorbani et al. offer four individual virtual offline sessions [33].

The remaining four programs offer primarily spiritual content and incorporate religious elements. In these programs, guided interviews are used to assess the participants’ experiences [26, 29], needs [40], quality of life [42], and coping strategies [29]. On this basis, individualized spiritual interventions are then offered, addressing themes such as hope [26, 29, 42], meaning of life [29, 40, 42], positive thinking [26, 42], self-esteem [40, 42], identity [40, 42], relationships [40], transcendence [40], and freedom [40]. The interventions comprise three [29], four [40], or five [26, 35] individual sessions, ranging from 30 [40], 45 [26] to 60 min [29, 35], and lasting four weeks [26], five weeks [35], six weeks [29], or 12 weeks [39].

(b) Faith community nursing:

Two studies examine the effects of an intervention based on Faith Community Nurse-led Cognitive Behavioral and Spiritual Counseling. As part of the intervention, a nurse trained in spiritual care visits the FCs at home and plans individual interventions for the FCs and their family members based on a health assessment. The interventions include promoting self-advocacy and self-care, relaxation training, reframing ineffective thinking, increasing assertiveness, incorporating pleasant daily activities, using religious coping strategies (e.g., prayer), meditation, positive affirmation, and communal support. The interventions are delivered through guided interviews and educational sessions [30, 41]. In the study by Kazmer et al., the FCs receive 12 individual sessions over 24 weeks [30], while in the study by Pomeroy et al., seven couple-based sessions are conducted over 12 weeks [41]. In both studies, the sessions last 60 min each [30, 41].

(c) Yoga:

All four yoga programs are couple-based. The FCs and their relatives in need of care learn individual and dyadic postures from an experienced yoga instructor during group sessions [25, 32, 38, 39]. In one study, participants join group sessions via Zoom due to the pandemic [32]. Milbury et al. also teach the FCs deep relaxation techniques, breath energization with sound resonance, and meditation in their programs [25, 32, 39]. In the program by Leng et al., the FCs and their relatives receive a total of 36 couple-based sessions over 12 weeks [38]; in the programs by Milbury et al., there are 15 group sessions over six weeks [25, 32, 39]. In all programs, sessions last 45 to 60 min. The FCs also receive printed materials [25, 39], pre-recorded instructions [38], or a DVD with the entire program [39], to practice daily yoga on their own at home [25, 32, 38, 39].

(d) Meditation:

The three meditation programs are conducted by an experienced instructor in group sessions [36, 37, 43], with one program accompanying dyads [37]. The programs include breathing techniques [36, 37, 43], body awareness exercises [36, 37, 43], exercises to promote mobility, flexibility, balance, strength, and endurance [36], connection to others and oneself [37], observing sensations, thoughts, and perceptions [36], and mindfulness in daily life [43]. Beauchamp et al. conduct four sessions of 60 min each for four weeks [37]; Diaz et al. conduct two sessions of 120 min each for four weeks [36]; and Waelde et al. support the FCs over 16 weeks with eleven sessions of 90 min each [43]. In addition to group sessions, the FCs should also complete the exercises at home [36, 37, 43].

(e) Mindfulness program:

Two programs offer mindfulness-based interventions for FCs, with one delivered entirely digitally via an app [31] and the other comprising group sessions and home-based exercises [27]. In both programs, the FCs are guided through mindfulness exercises (e.g., mindful eating, mindful stretching), body scans, and breathing exercises. In the group sessions, self-care and caring for others are also addressed [27], while the app focuses on anxiety, stress, acceptance, relationships, sleep, and recognizing emotions [31]. A total of four group sessions, each lasting 180 min, are held over four weeks [27]. The app is used by the FCs for at least 20 min a day over eight weeks (56 individual sessions) [31].

(f) Self-expressive therapies:

One study examines narrative self-expression. In this therapy, the FCs receive a single individual session. During this session, the FCs create a collage representing their life situation, guided by an art therapist who asks them questions about the collage. The session lasts 60 min [28].

### Outcomes of the interventions

The included studies are highly heterogeneous with regard to (a) the outcome measures used, (b) the reported intervention effects, and (c) the evaluation of intervention feasibility.

- Outcome measures:

A total of 61 different outcome measurement methods are identified, including standardized instruments, physiological parameters, and qualitative methods. The studies use an average of four instruments to measure the effectiveness of their intervention (range: 1 [34, 35] to 9 [31]). Most studies assess the burden on FCs in relation to stress (*n* = 10) [25–29, 31, 33, 34, 36, 38], depression (*n* = 7) [27, 31, 32, 36, 37, 39, 43], anxiety (*n* = 5) [27, 31, 36, 37, 40], fatigue (*n* = 3) [25, 31, 39], sleep disorder (*n* = 3) [25, 31, 39], and pain (*n* = 2) [31, 37]. General health (*n* = 7) [25, 29, 32, 38–41] and quality of life (*n* = 3) [31, 38, 40] are also frequently rated. Another focus is on spirituality, with studies examining both spiritual well-being (*n* = 7) [25, 27, 28, 33, 40–42] and spiritual and religious coping (*n* = 2) [29, 40]. Several studies also examine resources and positive coping mechanisms, including self-efficacy (*n* = 4) [26, 28, 35, 43], positive affects (*n* = 2) [28, 32], self-esteem (*n* = 1) [41], benefit finding (*n* = 1) [25], and post-traumatic growth (*n* = 1) [31]. Three studies also consider dyadic and social aspects, such as the relationship with the patient or perceived social support [25, 27, 38].

Aside from self-report data, three studies also integrate physiological markers to objectively measure stress responses [28, 36, 43]. These include measurements of heart rate variability [36], resting heart rate [36], blood pressure [36], cortisol [28, 43], and oxytocin [28].

Five studies use qualitative approaches to gain a deeper understanding of how the interventions are perceived [28–32]. In all qualitative studies, researchers collect data through interviews [28, 30–32] or open-ended questions in questionnaires [29]. In one study, researchers analyze the collages created by the FCs [28]; another study includes interviews with faith community nurses [30]; and another analyzes researchers’ interview notes [28]. An overview of all measurement methods is provided in Table 3.

(b) Reported effects:

The effects vary significantly depending on the type of intervention. In the case of spiritual training and therapy, the FCs report that the intervention helps them expand their knowledge and strategies to consciously use spirituality to cope with the caregiving situation [29]. Improvements are observed in self-efficacy [26, 35], positive spiritual/religious coping [29, 40], spiritual well-being [33, 40, 42], quality of life [29, 40], and mental health [29]. However, Perez et al. find that the improvements in spiritual well-being and spiritual/religious coping strategies are not consistent across all follow-up points [40]. Inconsistent results are observed regarding stress: while Bagheri et al. [26] and Mahdavi et al. [34] report a significant reduction, Qorbani et al. [33] find no change. Furthermore, Casaleiro et al. point out that physical health deteriorated [29].

The Faith Community Nursing intervention also shows significant improvements in spiritual well-being, self-esteem, and disease management, while no effects on depressive symptoms or health-related behaviors are observed [41]. Faith community nurses report that relaxation techniques help FCs address problems more structurally and calmly. FCs also report that the intervention helps them recognize the importance of their own health and teaches them skills to organize their time so they can practice self-care [30].

Among the yoga-based interventions, only two of four studies show statistically significant changes. These involve improvements in depressive symptoms [38], sleep quality [25, 38], and quality of life [38]. The remaining studies report non-significant effects, including improvements in depressive symptoms [25] and spiritual well-being [25, 39], with small to moderate effect sizes. At the same time, deteriorations in physical health and increased fatigue are observed [39]. Additionally, higher participation in yoga sessions is associated with significantly greater improvements [25], while differences between individual and dyadic sessions favor the individual format [32].

In contrast, none of the studies on meditation-based interventions find significant improvements in depressive symptoms [36, 37, 43] or self-efficacy [43]. Statistically significant effects are observed in terms of reduced anxiety [36, 37], improved life satisfaction [36, 43], and subjective sense of meaning [36], as well as physiological changes such as lower blood pressure [36], resting heart rate [36], heart rate variability [36], and diurnal cortisol slope [43].

The two studies on mindfulness-based interventions show contrasting results. While Kubo et al. find no statistically significant between-group effects for stress, anxiety, depression, sleep, pain, fatigue, or quality of life among FCs [31], Zhang et al. report statistically significant improvements in depression, experiential avoidance, family functioning, self-efficacy, and problem-solving coping [27].

The art-based intervention shows no effects and, rather, a tendency toward a decline in spiritual well-being, although the art therapist observes that the FCs engage in existential reflections on meaning and values, as well as on coping with grief and loss, and report reduced stress and reduced feelings of isolation [28].

(c) Feasibility of the interventions:

Seven studies examine the feasibility of spiritual interventions for FCs in a home setting [25, 29–32, 37, 39]. An average of 70% of FCs complete the intervention (range: 37% [37] to 100% [39]) [25, 31, 37, 39]. It becomes apparent that FCs attend sessions only irregularly and rarely perform independent exercises at home [25, 31, 32, 37, 39]. The main barriers to participation are lack of time due to caregiving responsibilities [25, 31, 32], changes in the patient’s health status [31, 32], and skepticism towards spiritual content [25, 29, 30]. Participation is higher when the intervention is perceived as relevant, and a strong relationship exists with the person conducting it [29, 30]. In particular, FCs who complete the intervention rate it as helpful [25, 31, 32, 39] and report that they will use the content even after the study had ended [31].

## Discussion

The 19 included studies show substantial heterogeneity in spiritual or religious interventions for FCs in the home setting. The interventions differ significantly in content, duration, and format, making it difficult to compare them and assess their effectiveness. Overall, the results show predominantly positive or neutral effects on the outcomes examined. Deteriorations are reported in only three studies, which primarily relate to physical health [29, 39], while one study finds a decrease in spiritual well-being [28]. When interpreting the results, it should be noted as a limitation that one study examines a yoga intervention for FCs of patients with high-grade glioma, so that confounding effects due to disease-related deterioration and its influence on the couple-based intervention cannot be ruled out [39]. The other interventions have a short total duration (1 session and 3 sessions of 60 min each) [28, 29].

Thus, the reported results suggest that spiritual interventions may initially lead FCs to become more aware of their own stress. If the intervention is too short, it seems only partially possible to teach sufficient coping strategies to actually reduce the stress they now recognize. Previous studies in this context also show that while FCs develop a greater awareness of their own needs and limits through spirituality [15–18], they are not able to immediately utilize spirituality as a helpful resource [18, 19]. Based on this, it can be concluded that spiritual interventions must be offered for a sufficiently long period to achieve positive effects. This finding is also supported by two reviews that identify significant improvements in FCs’ levels of stress, depression, anxiety, and self-efficacy through meditation and mindfulness-based interventions, though only when these interventions are tailored to and structured for the specific target group [44, 45].

On the other hand, prolonged interventions pose practical challenges. Most studies report difficulties with recruitment and rising dropout rates as the program duration increased [25, 27–29, 31, 32, 35–38, 40–43]. Participation is often irregular, and independent exercises are rarely integrated into daily life [25, 31, 32, 37, 39]. These challenges are also described in studies on FC recruitment. Common reasons cited include lack of time, a strong sense of responsibility toward patients, and a lack of interest [46, 47]. These findings emphasize that effectiveness depends not only on the intervention’s content but also on its feasibility in FCs’ daily lives. Therefore, interventions should be flexible in timing, easily accessible, and clearly tailored to the needs of the target group, because the more frequently FCs participate in such interventions, the greater is their impact on their outcomes, as demonstrated by Milbury et al. [25].

Another relevant aspect concerns the intervention format. Several of the included studies suggest that individual interventions offer benefits for FCs compared to dyadic approaches [25, 32, 38, 39]. While patients are more likely to attend joint sessions, FCs are more likely to avoid dyadic formats [25, 32, 39]. Accordingly, individual interventions tend to yield better outcomes for FCs [32]. One reason for this could be that FCs specifically seek space for their own needs and wish to focus more on their individual circumstances.

Furthermore, the relationship between FCs and the facilitator is a central factor in the effectiveness of these interventions. Qualitative findings highlight the importance of this relational aspect: trusting interactions not only strengthen the participants’ perceived level of support but also encourage them to continue attending the sessions [29, 30]. In more standardized or group-based formats, such as yoga or meditation programs, this aspect is less prominent [25, 31, 32, 37, 39], potentially reducing participants’ subjective relevance of the intervention.

Several studies suggest that nurses can play a key role in addressing this interpersonal aspect. The two studies on Faith Community Nursing included in this review offer an approach to linking relationship quality, spiritual competence, and structured intervention [30, 41]. In the U.S. context, faith community nurses are state-licensed nurses, typically holding bachelor´s or master´s degrees, and, after several years of clinical experience, they obtain specialized certification that encompasses the integration of professional nursing, spiritual care, health education, and community work within faith communities. They assume roles as health advisors and care coordinators with a focus on bio-psycho-social-spiritual health. Faith community nurses typically already have an established, trusting relationship with the community before they intervene [48, 49]. This structural difference distinguishes Faith Community Nursing interventions from other spiritual interventions in this review, in which such a relationship must first be established during the study. The present findings show that Faith Community Nursing interventions can lead to significant improvements in spiritual well-being, self-efficacy, and disease management [30, 41], with the qualitative study by Kazmer et al. [30] clearly highlighting that FCs perceive their relationship with the faith community nurses as a key factor in the intervention’s effectiveness. The Faith Community Nursing model thus illustrates that the structural integration of nursing professionals into communities can make spiritual interventions for FCs accessible, relationship-based, and sustainable; at least within the U.S. healthcare context, where faith community nurses are established as a specialized professional group. In other countries (e.g., Germany), no equivalent care model exists, limiting the generalizability of these findings and highlighting a care gap.

Implications for future research: Most studies on spiritual and religious interventions are pilot or feasibility studies, underscoring the need for randomized controlled trials with larger and more diverse samples. McAndrew et al. recommend conducting concurrent cost-benefit analyses to facilitate the long-term implementation of these interventions [50]. In general, to achieve the required sample size, the FCs should be more closely involved in the development of the interventions. Participatory and qualitative research approaches can help design interventions that are better tailored to needs and provide a better understanding of which spiritual and religious elements are perceived as particularly supportive.

Implications for practice: Spiritual and religious interventions should focus clearly on the needs of FCs and allow for individual experiences and reflection. Both individual and group settings can be effective, though supportive and trusting relationships play a central role in perceived effectiveness. Interventions should be designed to promote the development and application of coping strategies in everyday life over a sufficient period of time. Close coordination with FCs can help strengthen acceptance of and sustained use of the interventions.

### Strengths and limitations of the study

The study is based on a rigorous search strategy developed by a multidisciplinary team.

During the screening process, only interventions with a primary focus on spirituality and religion were included, while studies with only marginal spiritual and religious components were excluded. This enhances conceptual clarity but limits the generalizability to multimodal approaches. The included studies originate predominantly from the United States and Iran, which limits generalizability to other cultural and religious contexts. Most FCs were religious and/or spiritual, which may influence the results and limit their transferability to non-religious or non-spiritual populations. Furthermore, potential social desirability and cultural expectation biases should be considered. In some settings, such as Iran or parts of the US, particularly where religion plays a central societal role, participation in spiritual and religious interventions and the reporting of positive effects may be influenced by social norms or perceived expectations. Furthermore, although the review explicitly aimed to include both spiritual and religious interventions, a separate synthesis of religion-specific components and outcomes was not possible. Even studies using explicitly religious frameworks incorporated broader spiritual components that extend beyond a narrow sense of religiosity (e.g., mindfulness, self-care, or meaning-making). This conceptual overlap within the primary literature itself prevented a differentiated synthesis and reflects a broader challenge in this field of research. A quality assessment of the included studies was conducted, enabling a nuanced classification of the evidence. To the best of our knowledge, this is the most current scoping review on spiritual and religious interventions for FCs in the home setting.

## Conclusion

Spiritual and religious interventions can help FCs cope with the demands of home care. Positive effects are observed across several physical, psychosocial, and spiritual outcomes. However, these measures vary considerably in terms of their sustainability and scope. The effectiveness of these interventions depends on key factors, including duration, format, content, integration into daily life, the relationship with the individuals involved, and active participation. Future efforts should focus on developing structured, accessible, and FC-centered interventions that can be easily integrated into daily care. It may also be noted that the findings on faith community nursing point to a potential care gap in countries such as Germany, where no comparable model integrating spiritual care into community-based nursing currently appears to exist. Strengthening the evidence base through methodologically sound studies will be crucial for gaining a better understanding of the mechanisms of action and for promoting the integration of spiritual care into daily practice.

## Supplementary Information

Below is the link to the electronic supplementary material.

Supplementary Material 1

## Data Availability

The datasets used and/or analyzed during the current study are available from the corresponding author on reasonable request.

## Abbreviations

FC: Family caregiver
