# Supplementary material for: Caring for the caregivers: a scoping review of spiritual and religious interventions for family caregivers in home care
Source: BMC Nurs. 2026 Aug 3;25:691. doi: 10.1186/s12912-026-05173-y (PMC13445842; doi:10.1186/s12912-026-05173-y)
Supplement: Supplementary file 1 [file 12912_2026_5173_MOESM1_ESM.docx]

Appendix I: Search string for databases and Google Scholar

| Database | Search string | Number of records |
| --- | --- | --- |
| PubMed | (family caregiver* [MH] OR  informal caregiver* [MH] OR  spouse caregiver* [MH] OR  caregiver* [MH])  AND  (home OR  home care [MH] OR  domestic OR  outpatient)  AND  (spiritual* OR  spirituality [MH] OR  spiritualities [MH] OR  spiritual therapies [MH] OR  religion [MH] OR  faith healing [MH] OR  hope [MH] OR  meaning OR  purpose OR  existent* OR  transcendence* OR  connect* OR  aware* OR confidence OR creativ* OR  art [MH] OR  music [MH])  AND  (intervention* OR  coping OR coping skills [MH] OR  support OR counsel* OR  training* OR  education [MH] OR  instruction) | *n* = 153 |
| CINAHL | (family caregiver* OR  informal caregiver* OR  spouse caregiver*)  AND  (home OR  domestic OR  outpatient)  AND  (spiritual* OR  religion OR  faith)  AND  (intervention* OR  training* OR  education) | *n* = 28 |
| Cochrane | (family caregiver* OR  informal caregiver* OR  spouse caregiver*)  AND  (home OR  domestic OR  outpatient)  AND  (spiritual* OR  religion OR  faith)  AND  (intervention* OR  training* OR  education) | *n* = 17 |
| JBI | (family caregiver* OR  informal caregiver* OR  spouse caregiver* OR  caregiver*)  AND  (home OR  home care OR  domestic OR  outpatient)  AND  (spiritual* OR  spirituality OR  spiritualities OR  spiritual therapies OR  religion OR  faith healing OR  hope OR  meaning OR  purpose OR  existent* OR  transcendence* OR  connect* OR  aware* OR confidence OR creativ* OR  art OR  music)  AND  (intervention* OR  coping OR coping skills OR  support OR counsel* OR  training* OR  education OR  instruction) | *n* = 28 |
| PSYNDEX | (family caregiver* OR  informal caregiver* OR  spouse caregiver* OR  caregiver*)  AND  (home OR  home care OR  domestic OR  outpatient)  AND  (spiritual* OR  spirituality OR  spiritualities OR  spiritual therapies OR  religion OR  faith healing OR  hope OR  meaning OR  purpose OR  existent* OR  transcendence* OR  connect* OR  aware* OR confidence OR creativ* OR  art OR  music)  AND  (intervention* OR  coping OR coping skills OR  support OR counsel* OR  training* OR  education OR  instruction) | *n* = 24 |
| PsycINFO | (family caregiver* OR  informal caregiver* OR  spouse caregiver* OR  caregiver*)  AND  (home OR  home care OR  domestic OR  outpatient)  AND  (spiritual* OR  spirituality OR  spiritualities OR  spiritual therapies OR  religion OR  faith healing OR  hope OR  meaning OR  purpose OR  existent* OR  transcendence* OR  connect* OR  aware* OR confidence OR creativ* OR  art OR  music)  AND  (intervention* OR  coping OR coping skills OR  support OR counsel* OR  training* OR  education OR  instruction) | *n* = 33 |
| Scopus | (family caregiver* OR  informal caregiver* OR  spouse caregiver* OR  caregiver*)  AND  (home OR  home care OR  domestic OR  outpatient)  AND  (spiritual* OR  spirituality OR  spiritualities OR  spiritual therapies OR  religion OR  faith healing OR  hope OR  meaning OR  purpose OR  existent* OR  transcendence* OR  connect* OR  aware* OR confidence OR creativ* OR  art OR  music)  AND  (intervention* OR  coping OR coping skills OR  support OR counsel* OR  training* OR  education OR  instruction) | *n* = 2 |
| Springer Link | (family caregiver*)  AND  (home OR  outpatient)  AND  (spiritual* OR  religion)  AND  (intervention* OR  training* OR  education OR  instruction)  NOT  (review) | *n* = 60 |
| Web of Science | (family caregiver* OR  informal caregiver* OR  spouse caregiver* OR  caregiver*)  AND  (home OR  home care OR  domestic OR  outpatient)  AND  (spiritual* OR  spirituality OR  spiritualities OR  spiritual therapies OR  religion OR  faith healing OR  hope OR  meaning OR  purpose OR  existent* OR  transcendence* OR  connect* OR  aware* OR confidence OR creativ* OR  art OR  music)  AND  (intervention* OR  coping OR coping skills OR  support OR counsel* OR  training* OR  education OR  instruction) | *n* = 160 |
| Google Scholar | (family caregiver* OR  informal caregiver* OR  spouse caregiver* OR  caregiver*)  AND  (home OR  home care OR  domestic OR  outpatient)  AND  (spiritual* OR  spirituality OR  spiritualities OR  spiritual therapies OR  religion OR  faith healing OR  hope OR  meaning OR  purpose OR  existent* OR  transcendence* OR  connect* OR  aware* OR confidence OR creativ* OR  art OR  music)  AND  (intervention* OR  coping OR coping skills OR  support OR counsel* OR  training* OR  education OR  instruction) | *n* = 17.900  Only the first ten pages were scanned.  n = 103 |

Appendix II: Overview of all items in the qualitative studies (*n* = 3)

|  |  |
| --- | --- |
| 1. Question / objective sufficiently described?(*) | 4 |
| 2. Study design evident and appropriate? | 2.7 |
| 3. Context for the study clear? (*) | 4 |
| 4. Connection to a theoretical framework / wider body of knowledge? (*) | 2 |
| 5. Sampling strategy described, relevant and justified?(*) | 2 |
| 6. Data collection methods clearly described and systematic?(*) | 1.3 |
| 6. Data collection methods clearly described and systematic?(*) | 2.7 |
| 8. Use of verification procedure (s) to establish credibility? (*) | 2.7 |
| 9. Conclusions supported by the results?(*) | 1.3 |
| 10. Reflexivity of the account? | 0.6 |

Appendix III: Overview of all items in the quantitative studies (*n* = 18)

|  |  |
| --- | --- |
| 1. Question / objective sufficiently described? Yes(2)Partial(1)No(0) | 3.6 |
| 2. Study design evident and appropriate? Yes(2)Partial(1)No(0) | 2.9 |
| 3. Method of subject/comparison group selection or source of information/input variables described and appropriate? Yes(2)Partial(1)No(0) | 3.2 |
| 4. Subject (and comparison group, if applicable) characteristics sufficiently described? Yes(2)Partial(1)No(0) | 3.3 |
| 5. If interventional and random allocation was possible, was it described? Yes(2)Partial(1)No(0) | 3.8 |
| 6. If interventional and blinding of invesigators was possible, was it reported? Yes(2)Partial(1)No(0) | 1.1 |
| 7. If interventional and blinding of subjects was possible, was it reported? Yes(2)Partial(1)No(0) | 0.8 |
| 8. Outcome and (of applicable) exposure measure(s) well defined and robust to measurement / misclassification bias? Means of assessment reported? Yes(2)Partial(1)No(0) | 3.3 |
| 9. Sample size appropriate? Yes(2)Partial(1)No(0) | 1.4 |
| 10. Analytic methods described/justified and appropriate? Yes(2)Partial(1)No(0) | 3.3 |
| 11. Some estimate of variance is reported for the main results? Yes(2)Partial(1)No(0) | 3.3 |
| 12. Conrtolled for confounding? Yes(2)Partial(1)No(0) | 2 |
| 13. Results reported in sufficient detail? Yes(2)Partial(1)No(0) | 3.1 |
| 14. Conclusions supported by the results? Yes(2)Partial(1)No(0) | 2.9 |
